# Feeling of guilt explains why people react differently to resource depletion warnings

**DOI:** 10.1038/s41598-021-91472-0

**Published:** 2021-06-07

**Authors:** Thomas Baumgartner, Janek S. Lobmaier, Nicole Ruffieux, Daria Knoch

**Affiliations:** Department of Social Neuroscience and Social Psychology, Institute of Psychology, University of Bern, Fabrikstrasse 8, 3012 Bern, Switzerland

**Keywords:** Psychology, Human behaviour, Psychology and behaviour

## Abstract

Despite insistent warnings from climate scientists, the global environmental situation is further deteriorating. To date, only very few studies have investigated the impact of warnings on sustainable decision-making in controlled laboratory settings. Moreover, the few existing studies mainly looked at average warning reactions rather than taking individual differences into account. Here, we investigated individual differences in the reaction to resource depletion warnings and scrutinized the impact of emotions on behavioural changes by applying a resource dilemma task with warnings. Data-driven and model-free cluster analyses identified four different types of consumption behaviour. Importantly, guilt was positively related to sustainable decision-making after warnings. In contrast, a lack of guilt was associated with no behavioural change or even worse with more unsustainable behaviour after warnings. These findings contribute to the debate over effective climate change communication by demonstrating that issuing warnings about the climate crisis only leads to the intended behavioural changes if people experience guilt.

## Introduction

In 1992, the Union of Concerned Scientists and more than 1700 independent researchers warned that humans were on a serious collision course with the natural world. They proclaimed that fundamental changes were urgently needed to avoid the consequences our present course would bring. Twenty-five years later, Ripple and colleagues published a second warning to humankind, which was signed by more than 15,000 scientists from all around the world^1^. In this second notice, the scientists professed that in all but one measure (i.e., decline in ozone depleting substances) the global situation further deteriorated. So, for more than 30 years, the science has been clear: Mass extinction, collapsing ecosystem and rising temperatures are but a few of the inconvenient truths that humankind is facing. Yet, people react very differently to such warnings. While some carry on regardless, others have started to change their life-style and are calling for political and technical solutions. Why do some people react on these warnings while others do not?

To date, only very few studies have looked at the impact of warnings on sustainable decision-making. For example, Joireman et al.^2^ examined people’s harvesting behaviour in a hypothetical resource dilemma game and found that after participants were warned about depleting resources, size of harvests temporarily dropped. Importantly however, these authors only looked at average warning reactions and did not identify individual differences based on the harvesting behaviour. Yet, different people may react differently to the same situation. A first aim of the present study was to examine potential individual differences in sustainable behaviour after warnings.

Numerous theoretical approaches across various scientific disciplines have tried to explain sustainable decisions and behaviours with cognitive processes. While such cognitive models explain important aspects of human decisions and behaviour related to resource use, they fall short if the contributions of emotional processes are not considered^3^. Indeed, it has been shown that emotions can take active roles in promoting sustainable consumption choices^4–8^, but we know of no study that has investigated the role of emotions on sustainable behaviour after warnings. A second aim of the present research was hence to investigate whether emotions are related to sustainable behaviour after warnings, particularly the behavioural change after warning.

In the present study, we scrutinised whether people differ in their sustainable behaviour and how they change their behaviour after warnings. We then tested whether the emotional state can explain such individual differences. To do so, we employed a sequential resource dilemma task inspired by Joireman et al.^2^. In each round, participants decided how many points they would obtain from a public pool. Importantly, participants could translate their gained points into real money after the game. By deciding how much of the resource they would claim for themselves, participants are negotiating the internal conflict between their individual interests and the collective interests of the group (i.e., claiming a lot of the resource for themselves vs. constraining their individual resource consumption to avoid resource depletion). At two time-points, the participants were warned that the resources were seriously depleting, should they continue to obtain resources at the same rate. After the warning messages, participants were asked to rate how outraged, fearful, confident, guilty, powerless, and disappointed they felt. We chose these emotions based on the literature on sustainable behaviour and on our own considerations. “Fear” and “guilt” have often been used in studies concerning the role of emotions in sustainable choices^4–7,9^. “Powerless” was chosen to capture the feeling of helplessness, that one is not empowered to change the deteriorating state of the resources. “Confident” was chosen to measure the degree that the participant was “on top of things”, “disappointed” to capture the frustration about the deteriorating resources. “Outraged” was chosen to determine the feeling of fury and irateness about the decreasing resources. “Outrage” is a moral emotion^10^ which can be defined as anger provoked by the perception that a moral standard has been violated^11–13^.

We assume that people differ in the amount of resources they claim for themselves, with some people extracting more of the public goods while others behave more circumspect. With respect to reactions to warnings, we also expect individual differences. According to rational actor theories, some people may increase their resource consumption after being warned that resources are getting scarce, so that they can maximally benefit from the resource before it gets depleted^14^. Others may reduce their consumption after warnings in order to prevent resources from depleting^2,15^. In order to identify the full set of behavioural types that constitute the repertoire of extraction behaviour in our sample, we used a fully data-driven and model-free classification approach. This clustering approach divides participants into distinct behavioural types based on similarity/dissimilarity in their behaviour and automatically determines the optimal number of clusters (see methods section for details).We expect that the emotional state sparked by the warning will explain the distinct warning reactions of these behavioural types. Knowing more about the emotional processes underlying sustainable decision-making will help to understand why people react differently to warnings about the critical state of resources.

## Results

### Average warning reactions and behaviour variability

**Figure 1:**
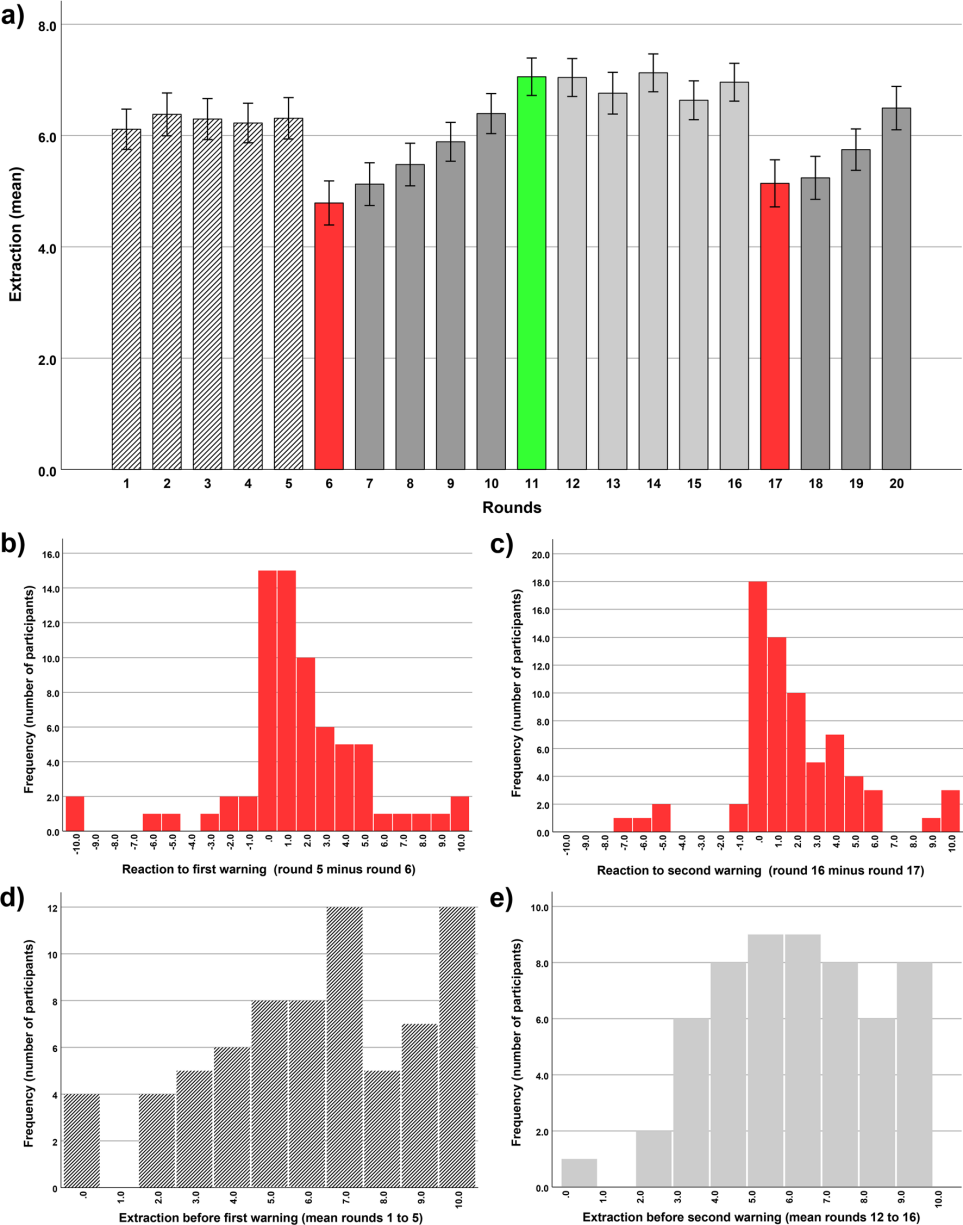
Average behaviour and variability in behaviour in the sequential resource dilemma. The bar graph in (**a**) illustrates the average behaviour across the 20 rounds of the sequential resource dilemma; the rounds after the first and second warning are depicted in red and the round after the indication that the situation is easing is depicted in green. Bar graphs in (**b**–**e**) depict histograms, demonstrating considerable variability in the first (**b**) and second (**c**) warning reaction and in the five rounds (mean) before the first (**d**) and second (**e**) warning. Note that warning reactions are calculated as differences between rounds immediately before the warnings and immediately after the warnings (round 5 minus round 6/round 16 minus round 17). Error bars depict standard errors of the mean.

The first set of analyses tested whether the warnings led on average to a decrease in extraction behaviour, as has been shown by a previous study^2^. For that purpose, we compared the rounds immediately before the warnings (rounds 5 and 16) with the rounds immediately following the warnings (rounds 6 and 17). Paired *t*-tests indicated that, on average, a highly significant decrease could be observed, both for the first warning (mean round 5 = 6.31, *SE* = 0.37, mean round 6 = 4.78, *SE* = 0.39, paired *t*-test: *t* = 3.66, *p* < 0.001) and the second warning (mean round 16 = 6.96, *SE* = 0.34, mean round 17 = 5.14, *SE* = 0.42, paired *t*-test: *t* = 4.83, *p* < 0.001, see Fig. 1a). Furthermore, we found that participants reacted, on average, to the indication that the situation was easing (after round 10, see method section for details) with an increase in extraction behaviour in the following round (mean round 10 = 6.39, *SE* = 0.36, mean round 11 = 7.05, *SE* = 0.33, paired *t*-test: *t* = − 2.66, *p* = 0.01, see Fig. 1a). However, this effect was less pronounced than the reactions to the warnings. Thus, on average, there are clear and strong reactions to warnings. Importantly, as shown in the histograms in Fig. 1b, c, there is considerable variability in the reactions to warnings, calculated and visualized as the difference between the rounds before the warnings (round 5 and 16) and the rounds after the warnings (round 6 and 17). Hereafter we label these differences as warning reaction scores. Some participants showed a strong decrease in extraction behaviour after the warning (positive value on the warning reaction score), while others did not react strongly to the warning (value around zero on the warning reaction score) or even showed an increase in extraction behaviour after the warning (negative value on the warning reaction score). Further, similar variability can be observed in the histogram for the rounds before the first warning (mean rounds 1–5, see Fig. 1d) and the rounds before the second warning (mean rounds 12–16, see Fig. 1e). Thus, the observed variability in these histograms suggest that there are distinct behavioural types.

### Emergence of the behavioural types

**Figure 2:**
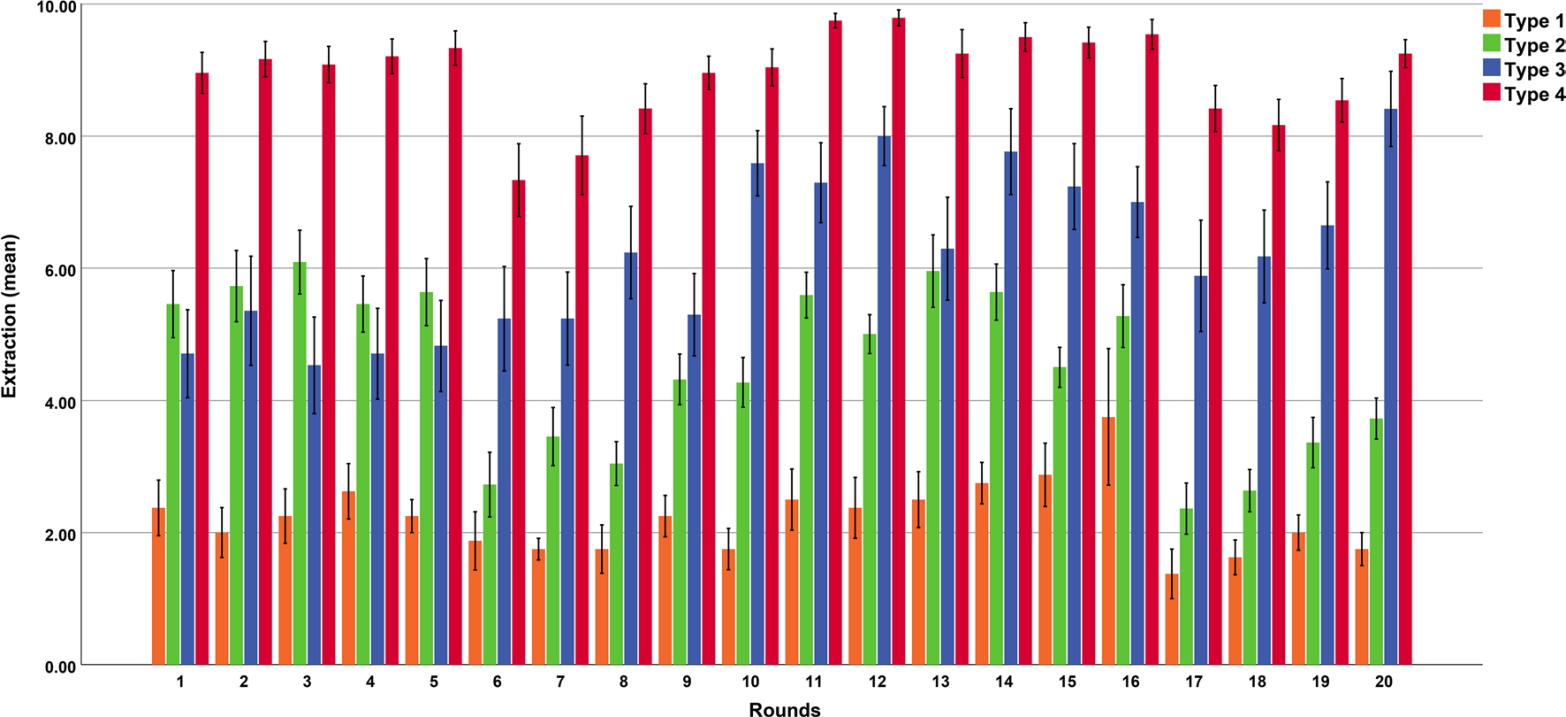
Distinct behavioural types in the sequential resource dilemma. Data-driven and model-free cluster analysis across all 20 rounds of the sequential resource dilemma identified four distinct behavioural types. Type 1 consisted of 8 participants, Type 2 of 22 participants, Type 3 of 17 participants and Type 4 of 24 participants. Note that we excluded Type 1 from further analyses because this type consisted of only 8 participants and showed a very sustainable behaviour across all rounds of the paradigm (before and after the warnings), leaving (almost) no margin and no necessity for warning reactions. Error bars depict standard errors of the mean.

In order to identify the behavioural types present in our study sample, we conducted a two-step cluster analysis (a data-driven and model-free approach, see methods section) of participants’ extraction behaviour, using all 20 rounds of the sequential paradigm. This analysis yielded a solution with four distinctive types, depicted in Fig. 2. Type 1 consisted of 8 participants (4 female, 4 male), Type 2 consisted of 22 participants (15 female, 7 male), Type 3 consisted of 17 participants (10 female, 7 male), and Type 4 consisted of 24 participants (18 female, 6 male). A visual inspection of Fig. 2 indicates that the four types showed a distinctive extraction behaviour in different stages of the sequential resource dilemma. There was one type (Type 1, consisting of only 8 participants) which demonstrated very low and sustainable extraction behaviour across all 20 rounds of the paradigm (mean rounds 1–20 = 2.21, SE = 0.27, see Type 1 in Fig. 2, orange bars). Although this type’s low extraction behaviour is important for making the world a more sustainable place, it is neither suited nor interesting for the research question of this study because it leaves almost no margin (and no necessity) for reacting to the warnings. Thus, in the following analyses, we focus on the other three types (Types 2, 3 and 4), which overall demonstrated a much less sustainable extraction behaviour than Type 1 (see Fig. 2), and therefore represent behavioural types to which environmental warnings apply. In the next step, we explore in detail in which of the distinct stages of the sequential paradigm the three types differed and whether these differences can be explained by distinct emotional reactions to warnings.

### Characterising the behavioural types

In order to analyse the behavioural differences between the three meaningful types (Types 2, 3 and 4, see above), we used univariate ANOVAs with between-subject factor behavioural types and subsequent post-hoc pairwise comparisons. We report these analyses in the sequential order of stages/events in the paradigm.

**Figure 3:**
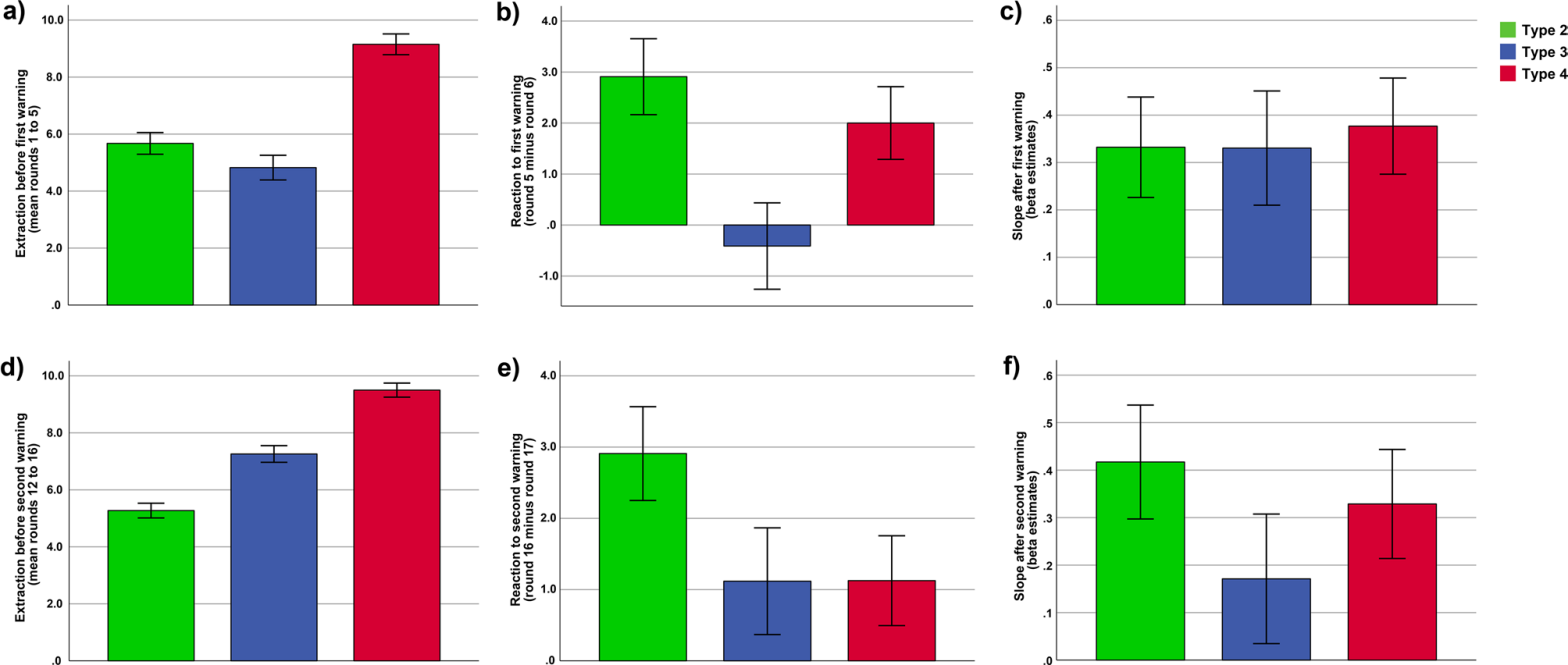
Extraction behaviour in the different stages of the sequential paradigm showed a characteristic pattern in the behavioural types. The bar graph in (**a**) illustrates the differences in extraction behaviour in the five rounds before the first warning (mean), which were qualified by higher extractions in type 4 compared to the two other types. The bar graph in (**b**) illustrates the differences in the reaction to the first warning, which were qualified by larger warning reactions (i.e. larger extraction differences between round 5 and round 6) in type 2 and type 4 compared to type 3. Note that positive values indicate a reduction in extraction after the warning, whereas values around zero indicate no change in extraction behaviour between the rounds before and after the warning. The bar graph in (**c**) illustrates the slopes in extraction behaviour in the rounds 6–10 following the first warning. All three behavioural types had a highly similar and positive slope, indicating a linear increase in extraction over time after the first warning. The bar graph in (**d**) illustrates the differences in extraction behaviour in the five rounds before the second warning (mean). Here type 4 demonstrated the highest extractions, type 2 the lowest extractions, with type 3 being in-between. Note that type 3 significantly increased the extraction behaviour in the rounds before the second warning (compared to the rounds before the first warning), whereas the other two types did not show such a change in extraction behaviour. The bar graph in (**e**) illustrates the differences in the reactions to second warning, which were qualified by (slightly) larger warning reactions (i.e. larger extraction differences between round 16 and round 17) in type 2 compared to the other two types. The bar graph in (**f**) illustrates the slope in extraction behaviour in the rounds 17–20 following the second warning. All three behavioural types had a similar (differences were not significant) and positive slope, indicating a linear increase in extraction over time after the second warning. Error bars depict standard errors of the mean.

In a first analysis, we checked whether the three behavioural types differed in the extraction behaviour in the five rounds before the first warning (rounds 1–5). Indeed, we found highly significant differences between the three behavioural types (*F*(2,60) = 35.61, *p* < 0.001, *η*_*p*_^2^ = 0.54, see Fig. 2 and Fig. 3a). Behavioural Type 4 demonstrated a very high extraction behaviour in the first five rounds (mean = 9.15, *SE* = 0.21), and this extraction behaviour was significantly higher compared to both other types (Type 2: *p* < 0.001; Type 3 = *p* < 0.001). Type 2 and Type 3 did not differ significantly (*p* = 0.146) and demonstrated a medium extraction behaviour in the first five rounds (Type 2: mean = 5.67, *SE* = 0.36; Type 3: mean = 4.82, *SE* = 0.63).

In a next analysis, we turned to the first warning reaction, calculated as difference between the round immediately before the warning and the round immediately after the warning (round 5 minus round 6, labelled as warning reaction score). Note that a positive value on this score indicates a reduction in extraction after the warning, whereas a negative value indicates an increase in extraction after the warning, and a value around zero indicates no change in extraction after the warning. Here the question arises whether the observed extraction behaviour before the first warning is related to the warning reaction. For example, do the two behavioural types which showed a highly similar extraction behaviour in the rounds before the warning (Type 2 and Type 3) also demonstrate similar warning reactions? And what about behavioural Type 4 which demonstrated a very high extraction behaviour in the first five rounds: Does this type ignore the warning or maybe even react particularly strong to the first warning? Univariate ANOVA revealed distinct warning reactions between the behavioural types (*F*(2,60) = 4.51, *p* = 0.015, *η*_*p*_^2^ = 0.13, see Figs. 2 and 3b). Post-hoc pairwise comparisons indicated that both Type 2 (warning reaction score: mean = 2.90, *SE* = 0.66, *t*-test versus zero: *p* < 0.001) and Type 4 (warning reaction score: mean = 2.00 , *SE* = 0.56, *t*-test versus zero: *p* = 0.002) demonstrated a pronounced warning reaction (i.e., a reduction in extraction behaviour in the round after the warning), with no differences between the two types (*p* = 0.381). In sharp contrast, Type 3 did not react at all to the warning (warning reaction score: mean = − 0.41, *SE* = 1.14, and *t*-test versus zero: *p* = 0.723). Notably, the warning reaction of Type 3 is significantly different from the other two types (Type 3 versus Type 2: *p* = 0.005, Type 3 versus Type 4: *p* = 0.033). These findings suggest that the reactions to the first warning are not related to the extraction behaviour demonstrated in the rounds before the warning. That is, behavioural types showing a similar extraction behaviour in the rounds before the first warning (Type 2 and 3) strongly differed in their warning reaction. Vice versa, behavioural types showing different extraction behaviour in the rounds before the warning (Type 2 and 4) demonstrated similar warning reactions.

Next, we turn to the rounds following the first warning (rounds 6–10, note that after round 10 participants received the information that the situation was easing). As can be seen in Fig. 1a, on average, people reacted to the warnings, but after a few rounds, they again reached their initial extraction level (before the warnings occurred). Thus, on average the warning reactions do not seem to be very sustainable. The more time passed since the warning, the closer people got to their initial extraction level. Here the question arises whether the two behavioural types (Type 2 and Type 4) which demonstrated a similar warning reaction (i.e. a reduction in extraction behaviour) also show a comparable extraction behaviour in the rounds after the warning (rounds 6–10) or whether Type 2 shows a more sustainable extraction behaviour than Type 4 in these rounds (or vice versa). And what about Type 3 which did not reduce the extraction behaviour after the warning: does this type continue to ignore the warning? In order to answer these questions, we used linear regression analyses to calculate in each individual participant the slope between the extraction behaviour in rounds 6–10 and the number of rounds since the warning had occurred (coded as 1–5). Note that a positive slope indicates an increase in extraction over time, a negative slope a reduction in extraction over time, and a slope around zero indicates no relation between extraction behaviour and the rounds that have passed since the first warning occurred. Univariate ANOVA revealed no differences between the three behavioural types in the slope after the first warning (*F*(2,60) = 0.062, *p* = 0.940, see Fig. 2 and Fig. 3c). Post hoc comparisons confirmed that all types had a highly similar slope (Type 2 versus Type 3: *p* = 0.992; Type 2 versus Type 4: *p* = 0.762; Type 3 versus Type 4: *p* = 0.770). Furthermore, the slopes of all behavioural types were positive (Type 2: mean = 0.33, *SE* = 0.13, Type 3: mean = 0.33, *SE* = 0.10, Type 4: mean = 0.37, *SE* = 0.08) and significantly different from zero (*t*-test versus zero: Type 2: *p* = 0.022; Type 3: *p* = 0.005; Type 4: *p* < 0.001), indicating that all behavioural types increased their extraction behaviour after the warning in a linear way. The more time had passed since the warning, the larger the extraction behaviour. These findings are particularly interesting regarding Type 3. Remember: Type 3 showed no reduction in extraction behaviour after the first warning (no warning reaction), but demonstrated a comparable slope as the other two types which reacted to the warning with a reduction in extraction behaviour. These findings therefore implicate that Type 3 considerably increased the extraction behaviour after the first warning. And indeed, comparing the five rounds before the first warning (Type 3: mean rounds 1–5 = 4.82, *SE* = 0.63) with the five rounds before the second warning (Type 3: mean rounds 12–16: 7.26, *SE* = 0.40) revealed that Type 3 significantly increased the extraction behaviour (paired *t*-test: *p* = 0.005). In contrast, Type 2 (mean rounds 1–5 = 5.67, *SE* = 0.36, mean rounds 12–16 = 5.27, *SE* = 0.24, paired *t*-test: *p* = 0.244) and Type 4 (mean rounds 1–5 = 9.15, *SE* = 0.21, mean rounds 12–16 = 9.50, *SE* = 0.17, paired *t*-test: *p* = 0.124) did not demonstrate a change in extraction behaviour between the five rounds before the first and second warning (see Figs. 2, 3a/d). Furthermore, univariate ANOVA revealed that the behavioural types significantly differed in extraction behaviour in the five rounds before the second warning (*F*(2,60) = 70.55, *p* < 0.001, *η*_*p*_^2^ = 0.70). Post hoc comparisons demonstrated that Type 4 had a higher extraction behaviour in these rounds compared to both other types (Type 4 versus Type 3: *p* < 0.001, Type 4 versus Type 2: *p* < 0.001) and Type 3 also had a significantly higher extraction behaviour compared to Type 2 (*p* < 0.001). Note that this last difference was not significant in the five rounds before the first warning (*p* = 0.146).

In a next analysis, we turned to the second warning reaction (after Round 16), again calculated as difference between the round immediately before the warning and the round immediately after the warning (round 16 minus round 17, labelled as warning reaction score). Univariate ANOVA did not show a difference in warning reactions between the behavioural types, but revealed only a trend (*F*(2,60) = 2.40, *p* < 0.010, *η*_*p*_^2^ = 0.07, see Fig. 2 and Fig. 3e). Post hoc comparisons indicated that Type 2 (warning reaction score: mean = 2.90, *SE* = 0.53) demonstrated a larger warning reaction than Type 3 (warning reaction score: mean = 1.11, *SE* = 1.10) and Type 4 (warning reaction score: mean = 1.13, *SE* = 0.44), however only at a trend level (Type 2 versus Type 3: *p* = 0.077; Type 2 versus Type 4 = 0.055). Type 3 and Type 4 did not differ in their warning reactions (*p* = 0.994). Thus, the differences between the behavioural types in this second warning reaction are less pronounced compared to the first warning reaction and Type 4’s second warning reaction seems to be more similar to Type 3’s warning reaction. However, note that both Type 2 and Type 4 demonstrate a significant warning reaction (one sample *t*-test versus zero, Type 2: *p* < 0.001, Type 4: *p* = 0.018), whereas Type 3’s warning reaction was not significantly different from zero (one sample *t*-test versus zero: *p* = 0.327). Comparing the two warning reactions with paired *t*-tests indicates that all three behavioural types showed rather similar warning reactions at the different time points (i.e. no significant differences between the first and second warning reactions), which is particularly true for Type 2 (paired *t*-test: Type 2: *p* = 1.00, Type 3: *p* = 0.269, Type 4: *p* = 0.209).

Finally, we also checked the slope after the second warning (rounds 17–20). Again, we used linear regression analyses to calculate in each individual participant the slope between the extraction behaviour in rounds 17–20 and the number of rounds since the warning has occurred (coded as 1–4). Findings revealed a similar pattern as for the analyses of the slopes after the first warning. Univariate ANOVA revealed no differences between the three behavioural types in the slope after the second warning (*F*(2,60) = 0.926, *p* = 0.402, see Figs. 2, 3f). Post hoc comparisons confirmed that all types had a similar slope (Type 2 versus Type 3: *p* = 0.181; Type 2 versus Type 4: *p* = 0.596; Type 3 versus Type 4: *p* = 0.380). Furthermore, the slopes of all behavioural types were positive (Type 2: mean = 0.41, *SE* = 0.11, Type 3: mean = 0.17, *SE* = 0.15, Type 4: mean = 0.33, *SE* = 0.11). However, only the slopes of Type 2 (*t*-test versus zero: *p* = 0.001) and Type 4 (*t*-test versus zero: *p* < 0.008) were significantly different from zero, which was not the case for Type 3 (*t*-test versus zero: *p* = 0.283), indicating that particularly Type 2 and Type 4 increased their extraction behaviour after the second warning in a linear way, that is, the more time has passed since the warning, the larger the extraction behaviour. We note that some caution may be warranted here because the paradigm ended shortly after the second warning and thus, there might not have been enough rounds to estimate these slopes properly.

Summing up, the analyses of the different stages and events of the sequential resource dilemma revealed a unique characterization of each behavioural type. One of the behavioural types (Type 2) was characterized by a moderate extraction behaviour before the two warnings, by consistent and strong warning reactions (i.e. reductions) after both warnings, and by a linear increase in extractions in the rounds following the warnings, reaching the initial extraction level again a few rounds later. Another behavioural type (Type 4) was characterized by a very high extraction behaviour before the two warnings, a strong warning reaction (i.e. reduction) after the first warning, which still existed, but was less pronounced after the second warning. Type 4 also demonstrated a linear increase in extraction in the rounds following the warnings, reaching the initial extraction level again a few rounds later. Finally, a last type (Type 3) was characterized by a moderate extraction behaviour before the first warning, no warning reaction (no reduction) after the first warning, and an increase of his extraction behaviour in the rounds following the first warning. Consequently, Type 3 reached a much higher extraction level in the rounds before the second warning, but still showed no pronounced warning reaction after the second warning.

### Behavioural types’ emotional reactions to the warnings

Finally, we examined whether differences in emotional reactions to the warnings help to explain the highly distinctive extraction behaviour of the three types in the different stages of the sequential paradigm. Participants reported after each of the two warnings how strongly they experienced each of the following six emotions (on a 7-point Likert scale, 0 = not at all, 3 = moderately, 6 = strongly): outrage, fear, confidence, guilt, powerlessness, disappointment.

**Figure 4:**
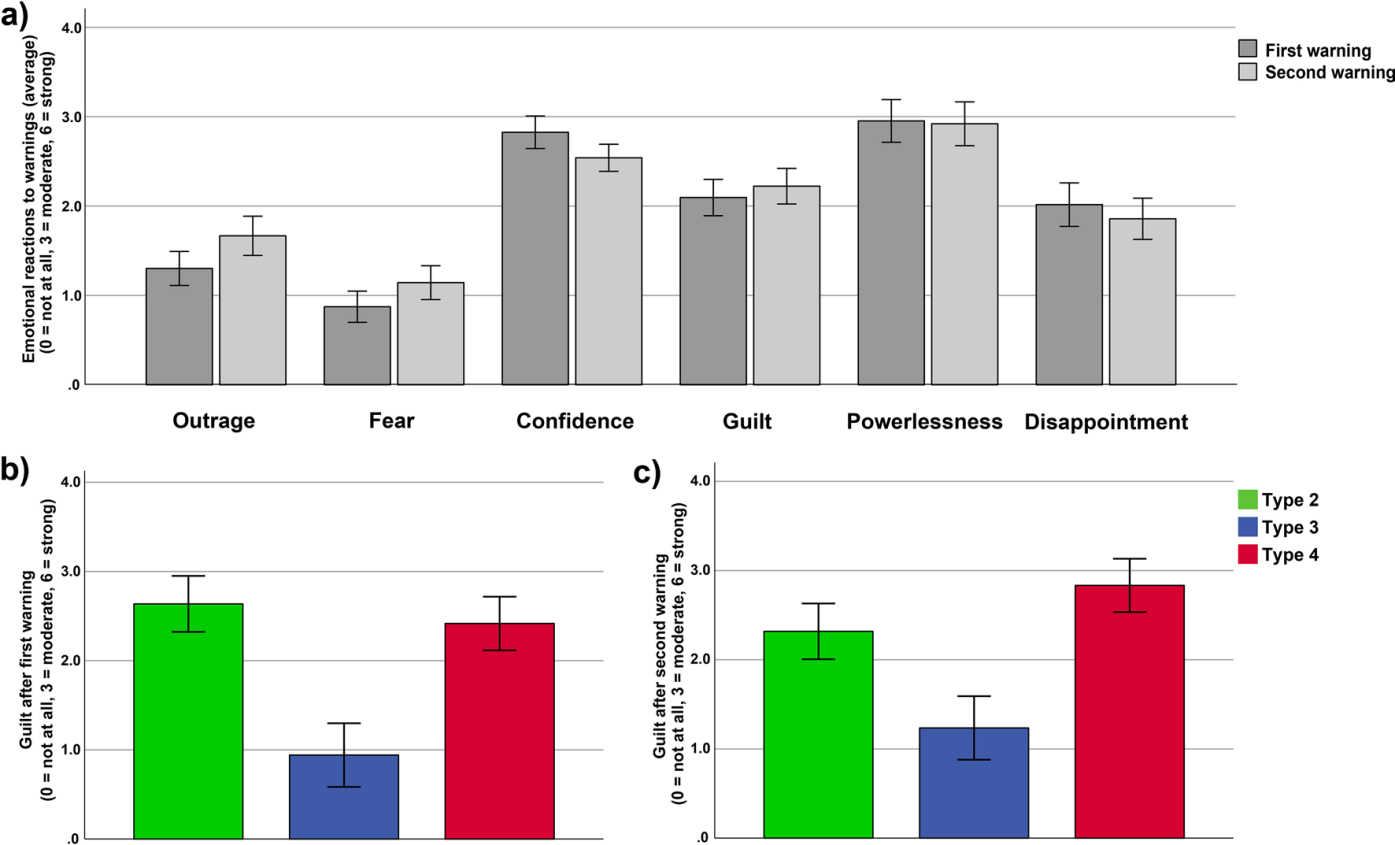
Average emotional reactions to the warnings and distinct emotional reactions of the three behavioural types. The bar graph in (**a**) illustrates the emotional reactions to the first and second warning, irrespective of behavioural types. On average, participants felt little outrage and fear, low to moderate levels of guilt and disappointment, and moderate levels of confidence and powerlessness after both the first and second warning. The bar graphs in (**b**) and (**c**) illustrate that type 2 and type 4 reported a moderate level of guilt after both warnings, which was significantly higher than the level of guilt reported by type 3, who felt only little guilt. Error bars depict standard errors of the mean.

On average, participants reported to have felt only little outrage (first warning: mean = 1.30, *SE* = 0.19, second warning: mean = 1.66, *SE* = 0.22) and fear (first warning: mean = 0.87, *SE* = 0.17, second warning: mean = 1.14, *SE* = 0.19), a low to moderate level of guilt (first warning: mean = 2.10, *SE* = 0.20, second warning: mean = 2.22, *SE* = 0.20) and disappointment (first warning: mean = 2.02, *SE* = 0.24, second warning: mean = 1.86, *SE* = 0.23), and a moderate level of confidence (first warning: mean = 2.82, *SE* = 0.18, second warning: mean = 2.54, *SE* = 0.15) and powerlessness (first warning: mean = 2.95, *SE* = 0.24, second warning: mean = 2.92, *SE* = 0.25). There was no difference between the emotional reactions after the first and second warning (paired *t*-test for each emotion: all *p*’s > 0.081, see Fig. 4a).

Interestingly, of these six emotions, only guilt revealed differences between the three behavioural types. We therefore focus on guilt in the main text of the manuscript. Please see Supplementary Table S1/S2 for detailed statistical information on all emotions.

We first analyzed the emotional reactions to the first warning, where the behavioural differences in warning reactions between the three types were more pronounced (as outlined in detail above). Univariate ANOVA revealed significant differences between the three behavioural types concerning guilt experienced after the first warning (*F*(2,60) = 7.29, *p* = 0.001, *η*_*p*_^2^ = 0.20, see Fig. 4b). Post hoc comparisons showed that the two types (Type 2 and Type 4) who reacted to the first warning, felt more guilty than Type 3 (Type 2 versus Type 3: *p* = 0.001; Type 4 versus Type 3: *p* = 0.002), who did not react to the first warning. Type 2 and Type 4 did not differ in the feeling of guilt (*p* = 0.615), and both reported a moderate level of guilt, whereas Type 3 reported to have felt only little guilt. Thus, a decrease in extraction behaviour after the first warning seems to be associated with the emotion of guilt.

Next, we analyzed whether guilt shows a similar or distinct pattern after the second warning. Univariate ANOVA revealed significant differences between the three behavioural types (*F*(2,60) = 5.90, *p* = 0.004, *η*_*p*_^2^ = 0.17, see Fig. 4c). Again, Type 2 and Type 4 reported a moderate level of guilt, whereas Type 3 only felt little guilt. The difference in guilt between Type 2 and Type 3 (*p* = 0.026) and between Type 4 and Type 3 (*p* = 0.001) were also significant, whereas no significant differences between Type 2 and Type 4 (*p* = 0.239) were observed. Thus, post hoc comparisons demonstrated a highly similar pattern of guilt after the second warning as after the first warning.

Taken together, guilt seems to be associated with behavioural change after warnings. Type 2 and Type 4, which both reacted to the warnings (more pronounced after the first warning), reported higher levels of guilt compared to Type 3, which showed no/little warning reactions and reported only little guilt. Furthermore, the extraction pattern after the first warning of Type 3 suggests that the lack of guilt has even more far-reaching detrimental consequences. Remember, Type 3 not only did not react to the first warning, but even worse, thereafter significantly increased the extraction and consequently reached a much higher extraction level in the rounds before the second warning compared to the rounds before the first warning (see in detail above).

## Discussion

We adopted a sequential resource dilemma task in which participants faced real monetary consequences. We investigated individual differences in sustainable behaviour after warnings and scrutinized the role of emotions in these behavioural changes. We found that after receiving a warning about depleting resources, people generally harvested less resources for themselves. Interestingly however, closer inspection of the data revealed four different types of behaviours. A very small group of people (Type 1) harvested so little right from the beginning that there was no necessity to further reduce their extraction after the warning. Because this highly sustainable group consisted of only 8 participants and consistently showed a very sustainable behaviour across all rounds of the paradigm (before and after the warnings), we refrain from discussing this group further in this paper. Others (Type 4) generally extracted a lot but decreased their consumption after the warnings. Again others (Type 2) showed moderate extraction levels in the beginning and also showed a decrease of consumption after warnings. A last group (Type 3) started with moderate extraction levels, but showed no decrease in extraction after warnings and even increased their extractions in the long run. Interestingly, emotions explained the different reactions to warnings. Specifically, feelings of guilt were related to the extraction behaviour after warnings: The two types that reduced their extraction after warnings both experienced guilt whereas the type that showed no warning reaction and thereafter increased their extraction levels experienced almost no guilt.

While previous studies suggest that people generally consume less when resources are scarce compared to when they are abundant cf.^16,17^, we demonstrate that not all people show this behaviour. Although the majority of participants dynamically reduced their consumption when resource scarcity increased (Types 2 and 4), a subset of participants (Type 3) did not react to the warnings and even increased their consumption of common goods when resources were in short supply. Notably, these different warning reactions did not depend on the initial extraction level: Type 3 participants did not differ from Type 2 participants in the beginning (i.e., before the first warning), but only showed differences in extraction behaviour after the warnings. Conversely, Type 2 and Type 4 showed very different extraction levels at the beginning, but both showed comparable warning reactions. In the rounds following the first warning, consumption of common resources increased again in all three groups, leading Type 3 to arrive at higher extraction levels in the rounds after the warning than in the rounds before while Types 2 and 4 returned back to the initial extraction levels.

What differentiates participants who react to the warnings in the intended direction from participants who do not reduce their extraction levels and even react with a backlash in the rounds after the warnings? It seems that the answer to this question can be found in the experience of guilt: Type 2 and Type 4 participants, who both reacted to the warnings, experienced feelings of guilt, while participants of Type 3, who did not react to the warnings and even increased their extractions in the long run, showed only very little guilt. These findings suggest that people who experience feelings of guilt after getting warned about depleting resources react with more sustainable behaviour, at least temporarily. Conversely, people who do not experience guilt do not reduce their extractions after the warning and even react in the opposite direction in the long run. So, while warnings indeed have the intended effect on sustainable behaviour in people who feel guilty, they seem to have unintended detrimental effects on people who experience little guilt.

The finding that guilt is a driving force behind behavioural changes after warnings is in line with research illustrating that emotions and affective processes contribute to adaptive decision-making^18,19^. Guilt has been shown to play a moderating role in other forms of sustainable decision-making. For example, when guilt was induced by environmental messages, participants’ intentions to repair environmental damages increased^20^. Another study found that the feeling of guilt, triggered by a social rule violation, motivated reparation and compensatory behaviour^21^. Other studies found that other emotions also take active roles in sustainable behaviour. For example, Skatova et al.^22^ showed in a repeated social dilemma game framed around shared electricity use at home that anger was damaging to cooperation as it lead to retaliation and an increase of defection. Feeling of guilt however repaired cooperation, resulting in higher levels of cooperation. Wang and Wu^6^ showed that pride, guilt, anger and respect had positive impacts on sustainable consumption choices of household appliances. Finally, van Zomeren et al.^9^ reported that environmental action intentions were increased by individuals' manipulated fear of the negative future consequences. Note that none of the previous studies reported here looked at the role of emotions in behavioural change specifically in response to warnings. We found that in the context of sustainable behaviour after warnings only guilt, but not outrage, fear, confidence, powerlessness, or disappointment, was related to changes toward more circumspect consumption.

We note that the current results do not necessarily imply a causal role of guilt in sustainable behaviour after warnings. Rather our findings reveal an association between feeling of guilt and warning reactions. Experience of guilt after the warnings helped to describe and differentiate the observed behavioural types that emerged from the cluster analyses. Furthermore, we used self-reported measures of the experienced emotions, which may be somewhat limited due to individual differences in reporting criteria. It would be interesting to see other studies corroborating our findings using causal manipulations and other types of emotional measures.

The present study aimed at investigating individual differences in the reaction to warnings and scrutinizing the impact of emotions on behavioural changes in the context of resource depletion. We identified four different behavioural types that are characterised by different extraction levels in the rounds before the warnings, by different reactions to the warnings and by different extraction levels in the rounds after the warnings. With regard to reactions to warnings, we found that guilt plays a key role in behaving more or less sustainably after warnings. Specifically, feeling guilty had a positive impact on the reactions to the warning, leading to more sustainable decision-making, regardless of the extraction levels before the warnings. In contrast, the lack of guilt lead people to react in the opposite direction and to extract more than before the warning. This means that warnings have a positive impact on people if they experience guilt. Conversely, warnings can actually have unintended detrimental effects in people who do not experience guilt. The present results yield important insights into the psychological mechanisms that can explain why people react so differently to warnings about depleting resources. Our findings suggest that issuing warnings about the climate crisis does not necessarily lead to the intended behavioural changes. People need to feel guilty in order to reduce their consumption.

## Methods

### Participants and procedure

We measured sustainable behaviour using a sequential resource dilemma game in 71 healthy university students from the University of Bern (47 women, 24 men, age: mean = 22.44; SD = 3.74). Students of economics, psychology and social sciences were excluded from participation to reduce the possibility of prior knowledge of the concept of resource dilemma games. We recruited participants for one academic semester in order to collect as many participants as possible during that time. Data were analyzed after the collection was complete. The study was approved by the ethics committee of the Faculty of Human Sciences at the University of Bern and was conducted according to the principles expressed in the Declaration of Helsinki. All participants gave written informed consent and were informed of their right to discontinue participation at any time. Participants received the equivalent of 25 USD for participating, in addition to the money earned in the resource dilemma game. Sustainable behaviour data was collected at a behavioural laboratory. Participants were tested in groups, one group of 15, two groups of 18 and one group of 20 people. Participants were randomly assigned to cubicles where they could make their decisions on individual computers in complete anonymity from the other participants.

### Sequential resource dilemma task

In each round of the resource dilemma task participants could harvest between 1 and 10 points (1 point = CHF 0.1) from a common pool. Participants were told that after each round, the pool would be partly refilled with a randomly chosen regeneration rate of either 10%, 15%, or 20% and that they would play the game for 30 rounds or until the pool was empty, whichever occurred first. They were also told that they would never know the exact size of the common pool, but that they would be informed, should the size of the pool change substantially.

In fact, all participants played 20 rounds. After Round 5 and Round 16, all participants received a warning message stating “The pool has diminished substantially. At this harvesting rate, the pool will be exhausted soon”. After Round 10, participants received an indication that the situation was easing and that the resources have replenished. After each warning, we additionally assessed participants’ emotions. Specifically, participants indicated on a 7-point Likert scale how outraged, fearful, confident, guilty, powerless, and disappointed they felt. We chose these emotions based on the literature on emotions and sustainable behaviour and on our own considerations (see introduction for details). After Round 20, participants received a message saying that the pool was exhausted and that the game would therefore terminate.

The participants' final payoff in the resource dilemma consisted of the total points they extracted from the common pool. Participants received written instructions and were asked control questions to ensure that they understood the task.

### Statistical analyses

In a first step, we tested whether the warnings lead (on average) to a subsequent decrease in extraction behaviour by contrasting the round immediately before the warnings with the round immediately after the warnings, using paired *t*-tests. Furthermore, we created histograms depicting the substantial individual variability in reactions to the warnings and in other stages of the paradigm (e.g., in the rounds before the warnings).

We then classified individuals into meaningful behavioural types based on their extraction behaviour in all 20 rounds of the sequential resource dilemma. For this purpose, we applied a two-step cluster analysis implemented in SPSS (Version 25.0) following a similar procedure as we described in^23^. This clustering procedure divides participants into different clusters/types based on similarity/dissimilarity in their behaviour. Importantly, this algorithm is naïve to the researchers’ assumptions about the number of clusters/types as it automatically determines the optimal number of clusters. The optimal number of clusters (i.e., the minimal number that best accounts for the variability in the data) is determined by a two-step procedure. The first step calculates the Schwarz-Bayesian information Criterion (BIC) for each number of clusters within a specified range and uses it to find the initial estimate for the number of clusters. The second step refines the initial estimate by finding the largest relative increase in distance between the two closest clusters in each clustering stage. The resulting statistics of the obtained cluster solution proved its good quality (silhouette measure of cohesion and separation = 0.6). Note that a silhouette measure above 0.5 corresponds to a good cluster solution.

In order to characterize the identified behavioural types, we analysed in which rounds/stages of the paradigm (e.g., rounds before the warnings, warning reactions, rounds after the warnings, for details see results section) the types demonstrated differences in extraction behaviour. We used univariate ANOVAs with the between-subject factor behavioural types and subsequent post-hoc pairwise comparisons (independent *t*-tests) to analyse the behavioural differences between the identified types. In addition, we used one-sample *t*-tests to examine whether the behavioural reactions (e.g., warning reactions) in each type were significantly different from zero. We report these analyses in the sequential order of rounds/stages in the paradigm.

Finally, we examined whether the emotional reactions to the warnings explain the distinct extraction behaviour of the behavioural types after the warnings (observed in the previous analyses). For that purpose, we again used univariate ANOVAs with between-subject factor behavioural types and subsequent post-hoc pairwise comparisons (independent *t*-tests). Such parametric analyses of Likert responses have been shown to be statistically robust, since it is assumed that Likert scales are symmetrical and equidistant^24^.

All statistical analyses were run with SPSS (version 25). Results were considered significant at the level of *p* < 0.05 (two-tailed). We use the abbreviation SE for standard error of the mean. As effect size measure partial eta^2^ (*η*_*p*_^2^) is reported, which is a measure of explained variance.

## Supplementary Information

**Supplementary Information 1.**

**Supplementary Information 2.**

## Data Availability

All data generated or analysed during this study are included as Supplementary Information file.
