# Supplementary material for: Feeling of guilt explains why people react differently to resource depletion warnings
Source: Sci Rep. 2021 Jun 7;11:11988. doi: 10.1038/s41598-021-91472-0 (PMC8185082; doi:10.1038/s41598-021-91472-0)
Supplement: Supplementary file 2 — Supplementary Information 2. [file 41598_2021_91472_MOESM2_ESM.docx]

Supplementary material for

**Feeling of guilt explains why people react differently to resource depletion warnings**

Thomas Baumgartner, Janek S. Lobmaier, Nicole Ruffieux & Daria Knoch

Department of Social Neuroscience and Social Psychology, Institute of Psychology, University of Bern, Bern, Switzerland

**This file includes:**

- Table S1. Statistical analyses of difference in emotional reactions to the first warning between the behavioral types
- Table S2: Statistical analyses of difference in emotional reactions to the second warning between the behavioral types

**Supplementary Table S1: Statistical analyses of difference in emotional reactions to the first warning between the behavioral types**

| Emotions  (first warning) | Univariate ANOVA | Type 2:  Mean  SE | Type 3:  Mean  SE | Type 4:  Mean  SE | Post hoc pairwise comparisons | | |
| --- | --- | --- | --- | --- | --- | --- | --- |
|  |  |  |  |  | Type 2 vs Type3 | Type 2 vs Type 4 | Type 3 vs Type 4 |
| Outrage | F = 0.072  P = 0.931  Eta^2^ = 0.002 | 1.36  0.31 | 1.35  0.38 | 1.20  0.31 | P = 0.983 | P = 0.733 | P = 0.767 |
| Fear | F = 0.389  P = 0.680  Eta^2^ = 0.013 | 1.04  0.36 | 0.64  0.24 | 0.87  0.27 | P = 0.381 | P = 0.681 | P = 0.609 |
| Confidence | F = 0.308  P = 0.736  Eta^2^ = 0.010 | 2.90  0.27 | 2.58  0.37 | 2.91  0.31 | P = 0.499 | P = 0.986 | P = 0.480 |
| **Guilt** | **F = 7.291**  **P = 0.001**  **Eta^2^ = 0.196** | 2.63  0.31 | 0.94  0.26 | 2.41  0.34 | **P = 0.001** | P = 0.615 | **P = 0.002** |
| Powerlessness | F = 0.138  P = 0.871  Eta^2^ = 0.005 | 3.09  0.31 | 2.76  0.44 | 2.95  0.46 | P = 0.601 | P == 0.816 | P = 0.752 |
| Disappointment | F = 0.571  P = 0.568  Eta^2^ = 0.019 | 2.31  0.42 | 2.05  0.49 | 1.70  0.37 | P = 0.681 | P = 0.292 | P = 0.572 |

Participants rated after the first warning how strongly they felt each of six emotions. Emotions were rated on a 7-point Likert scale (0 = not at all, 3 = moderately, 6 = strongly). Univariate ANOVAs revealed that only guilt showed differences between the three behavioral types. Significant ANOVA’s and significant post-hoc comparisons are depicted in bold.

| Emotions  (second warning) | Univariate ANOVA | Type 2:  Mean  SE | Type 3:  Mean  SE | Type 4:  Mean  SE | Post hoc pairwise comparisons | | |
| --- | --- | --- | --- | --- | --- | --- | --- |
|  |  |  |  |  | Type 2 vs Type3 | Type 2 vs Type 4 | Type 3 vs Type 4 |
| Outrage | F = 1.985  P = 0.146  Eta^2^ = 0.062 | 2.22  0.38 | 1.17  0.34 | 1.50  0.36 | P = 0.062 | P = 0.156 | P = 0.554 |
| Fear | F = 0.549  P = 0.580  Eta^2^ = 0.018 | 1.31  0.36 | 0.82  0.31 | 1.20  0.30 | P = 0.315 | P = 0.806 | P = 0.425 |
| Confidence | F = 1.316  P = 0.276  Eta^2^ = 0.042 | 2.54  0.19 | 2.17  0.29 | 2.79  0.28 | P = 0.343 | P = 0.488 | P = 0.110 |
| **Guilt** | **F = 5.975**  **P = 0.004**  **Eta^2^ = 0.166** | 2.31  0.28 | 1.23  0.32 | 2.83  0.33 | **P = 0.026** | P = 0.239 | **P = 0.001** |
| Powerlessness | F = 0.305  P = 0.738  Eta^2^ = 0.010 | 3.18  0.26 | 2.82  0.55 | 2.75  0.45 | P = 0.575 | P = 0.460 | P = 0.907 |
| Disappointment | F = 1. 375  P = 0.261  Eta^2^ = 0.044 | 2.31  0.36 | 1.35  0.38 | 1.79  0.42 | P = 0.106 | P = 0.331 | P = 0.450 |

**Supplementary Table S2: Statistical analyses of difference in emotional reactions to the second warning between the behavioral types**

Participants rated after the second warning how strongly they felt each of six emotions. Emotions were rated on a 7-point Likert scale (0 = not at all, 3 = moderately, 6 = strongly). Univariate ANOVAs revealed that only guilt showed differences between the three behavioral types. Significant ANOVA’s and significant post-hoc comparisons are depicted in bold.
